# On the importance of precision in cortical bone drilling: Integrating experimental validation and computational modeling

**DOI:** 10.1016/j.jor.2024.05.016

**Published:** 2024-05-13

**Authors:** Mohammadjavad (Matin) Einafshar, Mohadese Rajaeirad, Ahmad Babazadeh Ghazijahani, Michael Skipper Andersen

**Affiliations:** a Department of Material and Production, Aalborg University, Aalborg, Denmark; b Department of Biomedical Engineering, University of Isfahan, Isfahan, Iran; c Department of Biomedical Engineering, Amirkabir University of Technology, Tehran, Iran

**Keywords:** Controlled bone drilling, Excessive heat, Finite element prediction, Drill bit design, Cortical bone drilling kinematics

## Abstract

**Background:**

Cortical bone drilling is integral to orthopedic and dental surgeries, yet challenges such as thermal necrosis persist. Previous finite element (FE) models may overlook critical parameters, impacting accuracy. This study aims to integrate experimental and computational approaches to predict essential parameters—initial temperature, point angle, and spindle speed—enhancing precision in cortical bone drilling.

**Methods:**

Bovine cortical samples were utilized to systematically investigate the impact of four independent parameters on maximum temperature (MT) and maximum thrust force (MTF). Parameters included drill bit initial temperature (IT), diameter, point angle, and spindle speed (225–2700 rpm, feed rate 0.5–3 mm/s). Experimental procedures involved an orthopedic handpiece with titanium drill bits. DEFORM-3D V6.02 facilitated FE simulation, with the validated model developed for the second stage of the drilling process.

**Results:**

The validated model highlighted the significant impact of drill bit IT on MT, predicting a 26.14 % decrease in final bone temperature as IT decreased from 25 to 5 °C. Increasing the point angle from 70 to 120° resulted in a 13.1 % MT increase and a 26.9 % decrease in MTF. Spindle speed variations exhibited a 48.3 % temperature increase and an 82.8 % MTF decrease.

**Conclusions:**

Integrating experimental validation and computational modeling offers a comprehensive approach to predict drilling parameters. Precision in cortical bone drilling can be optimized by selecting specific parameters, including lower drill bit IT, smaller point angles, and controlled spindle speeds. This optimization reduces the risk of bone necrosis and thermal damage, thereby enhancing surgical outcomes.

## 1 Introduction

Bone machining, integral in treating various bone conditions, undergoes three stages: cutting, rimming, and drilling. Orthopedic surgeries, especially bone drilling, induce heat due to friction, risking thermal necrosis and compromising fixation and bone integrity^1^. Maintaining bone temperature below 45 °C is crucial to prevent complications.^2^^,^^3^ Previous research emphasizes the influence of temperature and thrust force on machining efficiency and the risk of thermal necrosis, detrimental to fixation and bone strength.^4^^,^^5^

With the increasing prevalence of robotic surgeries, precise data becomes paramount.^6^^,^^7^ Manual control of drill handpieces by surgeons often dictates drilling quality,^8^^,^^9^ but excessive forces can damage surrounding tissues and elevate the risk of cracks. These forces also generate heat, contributing to thermal necrosis.^2^^,^^3^ Additionally, thrust force and drill bit initial temperature (IT) are key factors exacerbating bone injury.^10^, ^11^, ^12^

Geometrical parameters, notably the point angle, greatly influence the drilling process. This angle, defining the relationship between the drill bit's cutting edges, profoundly impacts drilling efficiency and hole quality. Furthermore, the drill bit diameter affects the pilot hole size, drilling force and hence orthopedic and dental screw fixation.^1^, ^13^, ^14^, ^15^, ^16^, ^17^ Research by Einafshar et al. found that increasing the drill bit diameter from 2.5 to 3.2 mm led to a substantial 230 % reduction in maximum thrust force during drilling.^4^

Numerous studies have investigated the effects of different parameters experimentally, but conflicting findings exist among researchers. Alam et al.^12^ and Basiaga et al.^18^ suggest that higher spindle speeds reduce thrust force, whereas Lee et al.^19^ observed an increase. MacAvelia et al.^20^ found that increasing spindle speed reduces thrust force in human femurs but not artificial femurs. Thermal analyses on bone drilling also present conflicting conclusions, with Karaca et al.^21^ reporting temperature reduction with higher spindle speed, while Sharawy et al.^22^ reached the opposite conclusion.

Finite Element Analysis (FEA) is a potent tool for predicting bone machining outcomes, though its accuracy hinges on input parameter quality and may not fully capture tissue complexity. Tu et al.^23^ employed a 2D FE model to simulate drilling temperature increase but lacked experimental validation and force calculations. Childs et al.^24^ utilized a strain accumulation damage law and a metal machining FE model to predict forces and chip formation, with the material model being the primary factor affecting cutting force. Li et al.^25^ explored the drilling mechanism and found that feed rate, spindle speed, and drill diameter directly impact drilling temperature. Another FEM-based analysis established a safety zone for drilling parameters.^26^ Alam et al.^27^ demonstrated, through an FE thermo-mechanical study, that drilling speed has a greater influence on heat rise and thermal necrosis than feed rate.

While FEM enables exploration of material mechanical behavior, previous FE studies on bone drilling lack thorough consideration of IT and accurate material properties. This study aims to address these limitations by establishing an FE model for insight into bone drilling. Experimental tests were conducted to validate the model, which predicts effects of parameters challenging to implement experimentally, including initial temperature, point angle, and spindle speed, facilitating controlled drilling outcomes.

## 2 Methods

### 2.1 Experimental Methods

#### 2.1.1 Research design and specimen preparation

Two bovine femur pieces, aged 2–3 years, mimicking human bone properties, were used. Predominantly cortical, with an average thickness of 10 mm and a length of about 100 mm, sections were trimmed to fit the testing device. Obtained from a local butcher post-slaughter, no animals were sacrificed specifically for the study. Soft tissues were removed, and ten samples, each with around 50 drilling parts, were prepared following bone drilling guidelines.^28^ Stored at −20 °C in a saline solution of 30 % alcohol and water, samples were rasped to flatten the surface, akin to previous procedures.^4^

#### 2.1.2 Experimental setup

**Drilling device**: In the present study, an orthopedic drill handpiece (Iranian TajhizSina OR2013-02, Iran), featuring a dimmer for regulating spindle speed from zero to 900 rpm, was used. In this study we used a spindle speed of 900 rpm.^2^^,^^29^ The drilling handpiece was connected to the dynamic testing machine using an adjustor very similar to a previous study. Four screws and bolts were used to adjust the perpendicularity of the drill bit. A total of 28 orthopedic two-flute stainless steel drill bits with diameters of 2.5 mm and 3.2 mm were used. The point and helix angles were 70 and 25° as well as 80 and 35°, respectively. The drill bits were used no more than 20 times to ensure perfect drilling.

**Fig. 1:**
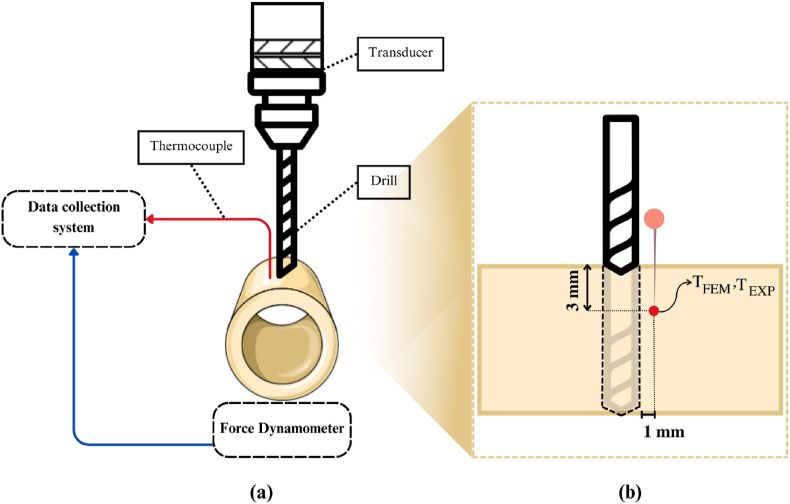
Schematic of (a) the drilling setup and process and (b) detailed position of the drill bit.

**Force and temperature recording device**: The drilling process and temperature recording followed procedures outlined in a recent paper.^4^ Measurement instruments included a Linear Variable Differential Transformer (LVDT) for displacement, a load cell for force, and a type K thermocouple with 0.1 °C sensitivity for temperature. As in prior studies, the thermocouple was positioned 3 mm deep near the drilling site within a 1-mm vicinity, following drilling of a small hole (Fig. 1).^4^^,^^30^

**Fig. 2:**
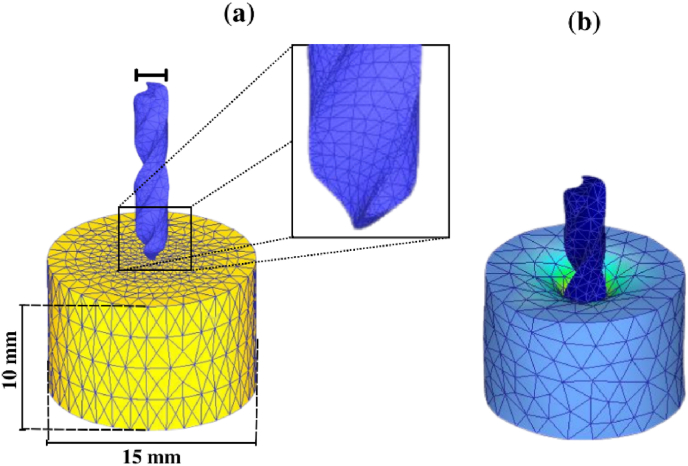
(a) Mesh structure of drill bit and workpiece (b) FE model during drilling.

**Fig. 3:**
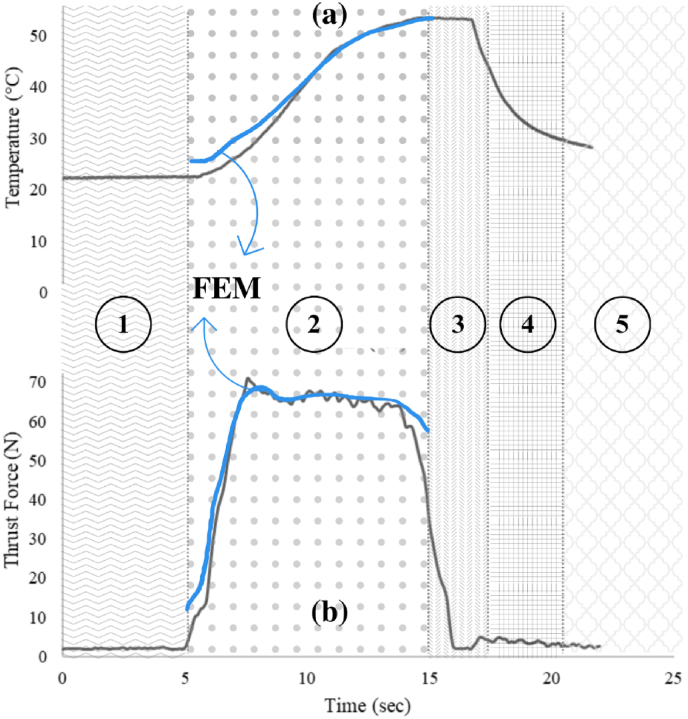
(a) Temperature versus time for drilling process with same feed rate (900 rpm), resting time, and exit rate. (b) Thrust force versus time for the drilling with the same specifications. All drilling processes include five stages: ① the initial stage where the drill bit descends but hasn't yet made contact with the bone, ② the drilling stage, ③ a resting stage, ④ the exit stage where the drill bit makes contact with the bone, and ⑤ the final exit stage where the drill bit exits the bone without making contact. The data provided in the figure are specified for a of 3.2 mm drill bit diameter.

**Fig. 4:**
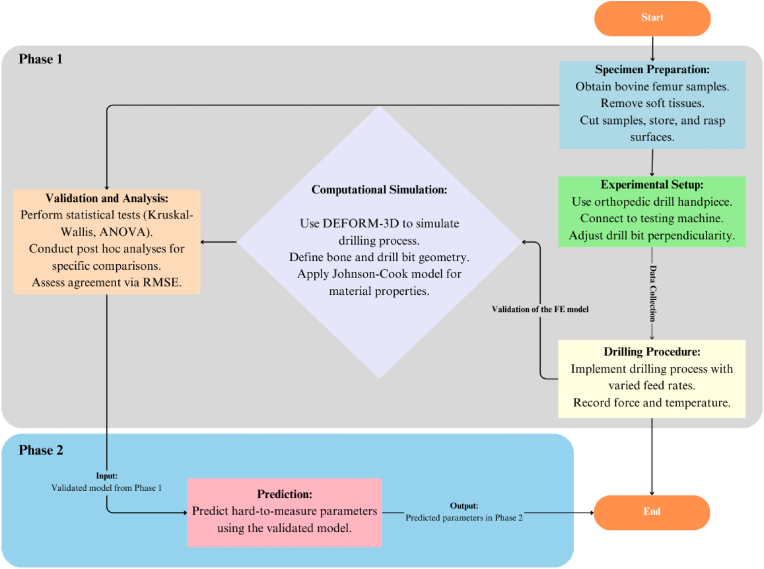
Block diagram illustrating the 2-phase methodology for the present study.

**Drilling procedure*:*** Feed rates of 0.5, 1, 1.5, 2, 2.5, and 3 mm/s were utilized in bone drilling across five stages: gradual descent to bone surface, drilling to 10 mm depth, resting, exiting bone, and surface exit (Fig. 3a and 3b). An illustration depicts a typical process with 1 mm/s feed rate, 1 s resting time, and 3 mm/s exit rate (Fig. 3)^3^ (see Fig. 4).

### 2.2 Computational simulation Methods

DEFORM-3D V6.02 (Scientific Forming Technologies Corporation, USA) was used to simulate and optimize the second stage of drilling, from initial bone penetration to full drilling depth (5–15 s in Fig. 3).

#### 2.2.1 Geometry and mesh

The cortical bone, modeled as a 15 mm diameter and 10 mm height cylinder (Fig. 2a), was meshed alongside the drill bit with specific design parameters matching experimental conditions. For a 2.5 mm diameter drill bit, a 70-degree point angle and 25-degree helix angle were assigned, while for a 3.2 mm diameter drill bit, an 80-degree point angle and 35-degree helix angle were established. Both workpiece and drill bit were finely meshed using tetrahedral elements, with radial gradient seeding to match interface meshes (Fig. 2a). Convergence analysis using seed sizes of 0.1, 0.2, 0.4, and 0.8 mm indicated convergence at 0.4 mm. Thus, a 0.4 mm seed size near the pilot hole was selected for subsequent analyses. Mesh updating (re-meshing) occurred after each simulation step due to element deletion during drilling, reaching deletion criteria (Fig. 2b).

#### 2.2.2 Material properties

Bone material properties were determined using stress and strain curves at different strain rates^28^ of the flow stress theory,^31^ employing the Johnson-Cook (JC) model known for its applicability in high strain, strain rate, and temperature conditions.^32^ This model accounts for strain-rate-dependent properties, plastic behavior, and thermal effects. Bovine bone in simulations had a thermal conductivity of 0.68 W/m°C and a thermal capacity of 1260 J/kg°C.^33^, ^34^

In drilling simulations, the drill bit's cutting edges operate at high speeds to shear the workpiece material, inducing chip formation for material separation. Finite element modeling in machining initially focused on a simplified parting line model, but the widely adopted criterion is the maximum plastic strain model. This model suggests material separation occurs when an element reaches critical plastic strain, and despite discussions on chip separation criteria, the maximum plastic strain model remains the most accepted method,^35^^,^^36^ widely implemented in drilling simulations.

Thermal, contact, and kinematic characteristics of the 3D FE model were defined, and the number of simulation steps and their durations were determined based on drilling depth. Thermal characteristics were inputted into DEFROM-3D software (Table 1), and experimental capture of maximum temperature near the drilling path at a 3 mm depth was conducted (Fig. 1).

(1) $$\sigma_{y}\left(\varepsilon_{p},{\dot{\varepsilon }}_{p},T\right)=\left[A+B{\left(\varepsilon_{p}\right)}^{n}\right][1+Cln\left({\dot{\varepsilon_{p}}}^{*}\right)\left[1-{\left(T^{*}\right)}^{m}\right]$$

**Table 1:** Thermal characteristics of the drill bit and bone.

Component	Thermal conductivity (W/m °C)	Thermal capacity (J/Kg °C)	Emissivity
Drill bit	15^47^	550	0.6
Bovine bone	0.68^32^	1260^30,31(p197)^	0.96^5^

#### 2.2.3 Loading and boundary condition

The IT of both the drill bit and bone was set to 25 °C to replicate experimental conditions. The bone was fully fixed, and a friction coefficient of 0.6 was applied at the drill bit-bone contact surface. Spindle speeds ranged from 225 to 2700 rpm, and feed rates ranged from 0.5 to 3 mm/s to simulate drilling kinematics.

#### 2.2.4 Criteria for validation and statistical analysis

The study categorized parameters into three groups: independent variables (feed rate in Phase I, and IT, point angle, and drill bit diameter in Phase II), dependent variables (MT and MTF), and constant parameters (IT of drill bit and bone, drill bit diameter, bone samples, bone quality, point angle, spindle speed).

The investigation focused on drilling speeds (0.5, 1, 1.5, 2, 2.5, 3 mm/s) and their influence on temperature, force observed in drill bit with 3.2 mm and 2.5 mm diameter, with a sample size of 45 in each speed group (N = 270) using SPSS v.27.

In order to assess the agreement between the FE simulations and experimental data, the root mean square error (RMSE) method was utilized (Eq. (2)).

(2) $$\text{RMSE}=\sqrt{\frac{\sum_{i=1}^{N}{\left\|y\left(i\right)-\hat{y}\left(i\right)\right\|}^{2}}{N}}$$

where *N* represents the total number of observations, y(i) denotes the actual values, and $\hat{y}\left(i\right)$ represents the predicted values.

## 3 Results

The Kruskal-Wallis test revealed a significant relationship between feed rate and temperature (p < 0.001, eta square = 0.693), indicating a large effect size. One-way ANOVA demonstrated a statistically significant relationship, with large effect sizes (eta square = 0.897 and 0.658 for 3.2 and 2.5 mm diameters, respectively), between feed rate and forces,^37^ emphasizing feed rate's pivotal role in temperature and force variations during machining. Pairwise comparisons with a confidence interval of 95 % for forces 3.2 and 2.5 revealed non-significant p-values (>0.05), while all other comparisons showed high significance (p < 0.001), rejecting the null hypothesis.

### 3.1 Phase Ⅰ: validation of the FE model

**Fig. 5:**
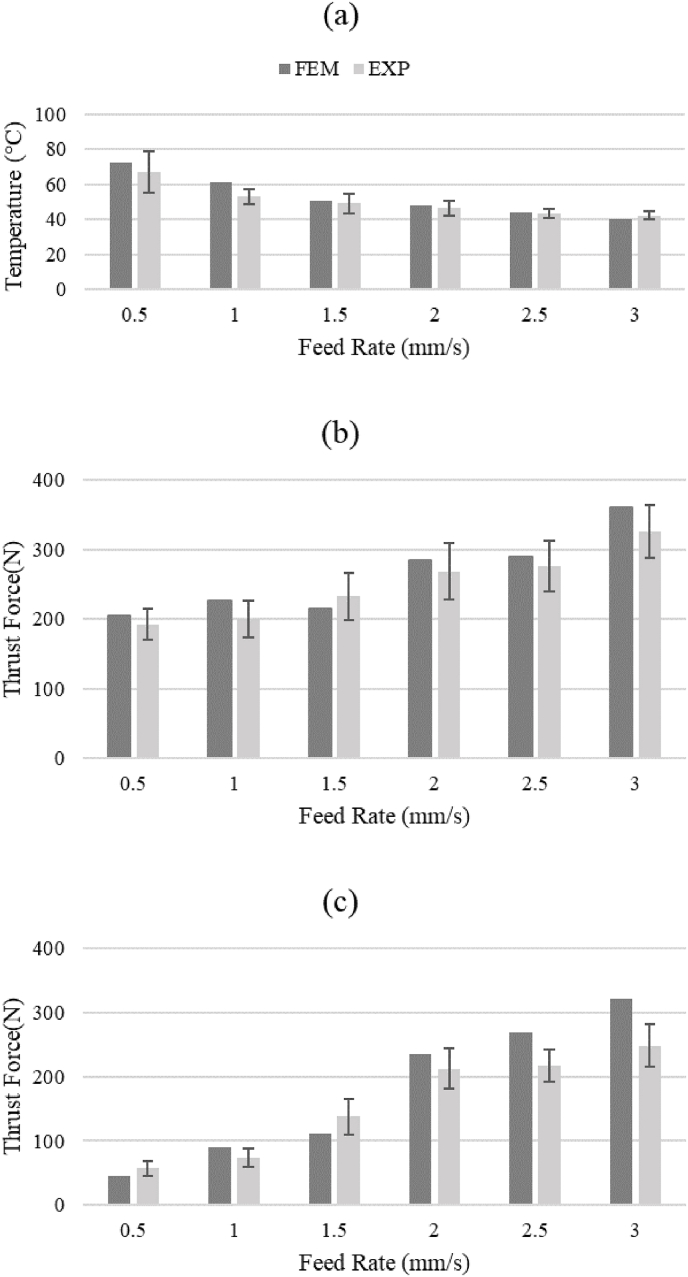
Validation of the FE model with experimental data (a) Temperature vs. feed rate (drill bit diameter of 3.2 mm), (b) thrust force vs. feed rates (drill bit diameter of 2.5 mm), and (c) thrust force vs. feed rates (drill bit diameter 3.2 mm).

Temperature-time curves were recorded for each experimental group, with average peak temperatures reported every five tests (Fig. 3a and Fig. 5 a). The feed rate of 0.5 mm/s showed the highest peak temperature, significantly different from other groups (P < 0.001), while the 3 mm/s group exhibited the lowest peak temperature, demonstrating an inverse relationship between temperature and feed rate. Temperature curves depicted a decline as the feed rate increased (Fig. 5a). The coefficient of variation (CV) for maximum temperature (MT) and maximum thrust force (MTF) mean values were 9.64 % and 16.47 %, respectively, indicating considerable variability within the data.

In the second stage, maximum thrust force during drilling was determined (Fig. 5b and 5c).^12^ For a 2.5 mm diameter drill bit, the highest thrust force occurred at a feed rate of 3 mm/s, while the lowest was at 0.5 mm/s. This pattern also held for a 3.2 mm diameter drill bit.

### 3.2 Phase Ⅱ: Prediction of the Interested parameters

**Fig. 6:**
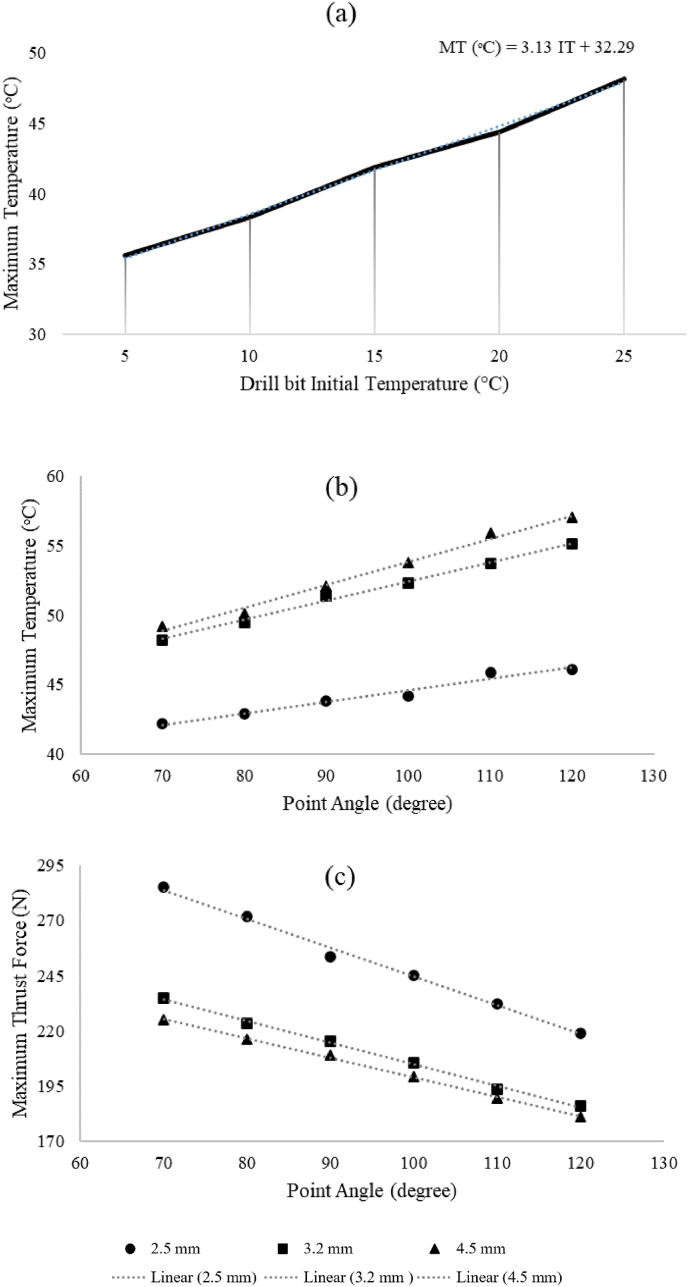
Prediction of bone's maximum temperature (MT) (ᵒC) at the end of stage 2 versus (a) initial temperature of the drill bit and (b) point angle and drill bit diameter. (c) The predicted values of maximum trust force (MTF) (N) vs. point angle and drill bit diameter.

The influence of drill bit initial temperature (IT) on bone temperature (MT) was studied while maintaining constant kinematic parameters (spindle speed = 900 rpm, feed rate = 2 mm/s, point angle = 80, helix angle = 35) (Fig. 6a). A linear correlation was observed between drill bit IT and bone temperature.

The validated FE model predicted the effect of point angle and drill bit diameter (2.5, 3.2, and 4.5 mm) on MT (Fig. 6b) and MTF (Fig. 6c). Increasing the point angle by 50° resulted in temperature increases of 3.9 °C, 6.9 °C, and 7.8 °C for drill bit diameters of 2.5 mm, 3.2 mm, and 4.5 mm, respectively, in MT. Conversely, MTF decreased by 66 N, 49 N, and 44 N for the same drill bit diameters with increasing point angle.

**Fig. 7:**
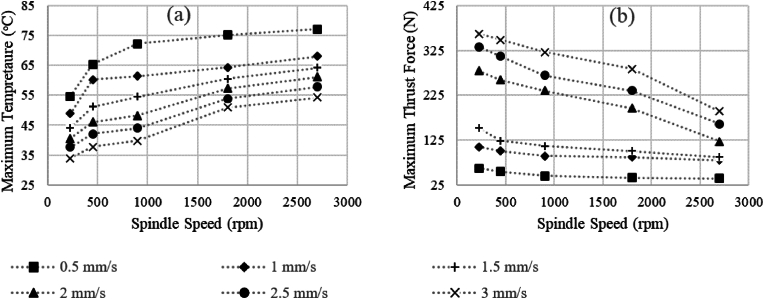
Predicted values for (a) maximum temperature (MT) and (b) maximum thrust force (MTF) versus spindle speed and feed rates of 0.5–3 mm/s. The values of diameter, point and helix angle are constant and equal to 3.2 mm, 70° and 25°, respectively.

Spindle speeds ranging from 225 to 2700 rpm were analyzed for their impact on MT and MTF (Fig. 7). Results showed a significant increase in MT by an average of 48.3 %, and a decrease in MTF by an average of 82.8 % with increasing spindle speed.

## 4 DICUSSION

Controlled bone drilling requires careful consideration of kinetic and kinematic parameters,^8^^,^^10^^,^^29^ impacting accuracy and precision. Kinetic parameters involve forces and moments affecting drilling, while kinematic parameters influence drilling quality and bone damage.^38^^,^^39^ Accurate assessment, crucial for evaluating robotic systems and optimizing drilling, can be achieved through computational techniques like FEA.^40^

In the initial stage, the FE model was validated using experimental data, showing consistency with previous findings.^41^ Increasing feed rate during drilling resulted in a temperature decrease (Fig. 5a).^4^^,^^42^, ^43^, ^44^, ^45^ The RMSE value of 4.29 °C indicates strong agreement between the FE model and experimental results.

Increasing feed rate led to higher thrust forces for both 2.5 mm and 3.2 mm drill bit diameters, with the increase more pronounced for the latter. The highest thrust force occurred with the 2.5 mm drill bit at a feed rate of 3 mm/s. RMSE values of 21.5 N and 39.8 N were obtained for the 2.5 mm and 3.2 mm drill bit diameters, respectively, confirming agreement between experimental and FE results. MTF increased by 69 % and 335 % for the 2.5 mm and 3.2 mm drill bit diameters, respectively, as feed rate increased from 0.5 to 3 mm/s, consistent with previous studies.^4^^,^^11^^,^^19^ Similarly, the FE model showed an increase of 76 % and 613 % in MTF for the 2.5 mm and 3.2 mm drill bit diameters, respectively, over the same feed rate range. The decrease in thrust force for larger drill bit diameters is attributed to their greater cutting surface area.^8^

Controlling temperature during bone drilling is critical to prevent thermal damage and optimize healing.^38^^,^^39^ The validated FE model^45^ confirms a direct correlation between initial temperature (IT) and maximum temperature (MT). A decrease in IT by 20° results in a notable 26.14 % decrease in MT, highlighting the importance of reducing IT to mitigate thermal risks and potential necrosis. Maintaining IT below 20 °C can keep bone temperature below the safe threshold of 45 °C, crucial for preventing significant damage.^2^^,^^3^^,^^26^ Further investigations are needed to explore the impact of IT and assess enhanced cooling drill bits.

MT in bone drilling is significantly influenced by the point angle, with larger angles leading to higher values due to increased shear deformations and heat generation.^41^ A 50-degree increase in point angle results in a 13.1 % average increase in MT but a 26.9 % average decrease in MTF (Fig. 6b and Fig. 6c).^43^^,^^46^ This is attributed to the inverse relationship between point angle and contact angle, making cutting action easier but increasing frictional forces and temperature rise in the bone.^41^ Larger drill bits with larger diameters act as cooling elements, produce larger bone chips, and facilitate heat removal, resulting in relatively smaller temperature shifts compared to smaller diameters.

Temperature rise during machining decreases with increased feed rate while maintaining constant spindle speed (Fig. 7a), indicating reduced specific heat generation. Conversely, increasing spindle speed leads to higher heat generation and temperature rise at maintained feed rates.^21^^,^^44^^,^^47^ The influence of spindle speed on drilling forces varies among studies, with conflicting findings.^27^^,^^41^^,^^44^^,^^47^ Some show a decrease in maximum thrust force (MTF) with increased spindle speed,^2^^,^^12^^,^^18^ while others report contrasting or limited effects.^10^^,^^47^ Comparing results from Fig. 6b and Fig. 7 a suggests that the effect of increasing spindle speed on temperature rise is more significant than diameter changes.^2^

Increasing spindle speed during drilling impacts chip size, exit conditions, and resulting forces significantly.^4^^,^^42^ Higher spindle speeds lead to smaller chip sizes, improving chip exit conditions and reducing force values (Fig. 7b). This reduction in force lowers the risk of drill fracture and assists surgeons during drilling.

The study concludes that reducing MT in bone drilling is achievable by selecting larger feed rates, lower initial temperatures (IT) of the drill bit, smaller point angles, smaller drill bit diameters (if feasible), and lower spindle speeds. Validation results confirm alignment between predicted and experimental outcomes across various drilling conditions, offering the potential to prevent bone fracture and drill bit breakage through real-time control of feed rate and spindle speed simultaneously.

A potential limitation of the study is the use of constant values for exit rate and resting time, although previous research suggests these factors have minimal impact on thrust forces during drilling.^4^ Ensuring the freshness of bovine bone samples was addressed by segmenting bones to minimize the use of non-fresh specimens. Another limitation is the use of bovine bone samples, which may not directly translate to human bone due to density differences.^10^^,^^29^ Future investigations could utilize human cadaveric samples for more accurate conclusions. Nonetheless, the study's primary aim was to evaluate individual parameters in bone drilling to enhance overall process quality.

## 5 Conclusion

In conclusion, our bi-phase study successfully utilized finite element (FE) modeling to predict crucial parameters in cortical bone drilling, offering a cost-effective alternative to traditional experimental approaches. The findings highlight the importance of higher spindle speeds in reducing thermal damage and bone breakage risk. While our study illuminates the impact of various parameters on drilling outcomes, future research could employ human cadaveric samples for more accurate conclusions. Despite limitations, our study's primary goal was to evaluate individual parameters to enhance bone drilling process quality.

## FUNDING

This research did not receive any specific grant from funding agencies in the public, commercial or not-for-profit sectors.

## Ethical approval and patient consent

The study used bovine bone samples taken from a local butcher, thus no IRB approval or patient consent was required.

## Declaration of interest letter

We affirm that there are no conflicts of interest associated with this research work. None of the authors have financial or personal relationships that may have influenced the results or interpretations presented in this manuscript.

## Ethical Statement

We confirm that the bovine bone samples used in this study were obtained from a local butcher shop and no animals were purposefully harmed or killed for the purpose of this research. We uphold ethical standards in our work and ensure that all research involving animals is conducted with the utmost care and respect for animal welfare.

## Guardian/patient's consent

The study used bovine bone samples taken from a local butcher, thus no IRB approval or patient consent was required.

## CRediT authorship contribution statement

**Mohammadjavad (Matin) Einafshar:** Conceptualization, Methodology, Writing – original draft, Writing – review & editing, Project administration, Supervision. **Mohadese Rajaeirad:** Data curation, Writing – original draft, Visualization, Investigation, Validation, Writing – review & editing. **Ahmad Babazadeh Ghazijahani:** Software, Data curation. **Michael Skipper Andersen:** Writing – review & editing.
